# Editorial: Stress and Neurodevelopment

**DOI:** 10.3389/fnins.2022.898872

**Published:** 2022-04-13

**Authors:** Panagiota Pervanidou, Agorastos Agorastos, George P. Chrousos

**Affiliations:** ^1^Unit of Developmental and Behavioral Pediatrics, First Department of Pediatrics, School of Medicine, “Aghia Sophia” Children's Hospital, University of Athens, Athens, Greece; ^2^2nd Department of Psychiatry, School of Medicine, Faculty of Health Sciences, Aristotle University of Thessaloniki, Thessaloniki, Greece; ^3^University Research Institute of Maternal and Child Health and Precision Medicine, Medical School, “Aghia Sophia” Children's Hospital, National and Kapodistrian University of Athens, Athens, Greece

**Keywords:** stress, neurodevelopment, prenatal, autism, ADHD, trauma, early life stress (ELS), prenatal maternal stress (PNMS)

Stress is defined as the state of threatened homeostasis. It has been associated with dysregulation of the stress system, consisting of the Hypothalamic-Pituitary-Adrenal (HPA) Axis and the Locus Caeruleus-Norepinephrine/Autonomic Nervous Systems (LC-NE/ANS), as evidenced by altered central and peripheral biomarkers of this system (Chrousos and Pervanidou, 2014). Experimental and clinical studies have shown that early life stress, particularly prenatal and early childhood stress (e.g., malnutrition, psychological stress and trauma, physical illness, etc.) may have pervasive and persistent effects on frontal cortical- hypothalamic, and mesolimbic circuits, including the amygdala, the hippocampus and the reward system. These structural and functional alterations during brain development may be associated with later neurodevelopmental and neurobehavioural symptoms and disorders. Although the existing research has connected early life stressors or traumatic experiences to changes in brain development and to related adverse outcomes, the underlying pathophysiological mechanisms are not fully understood as yet (Agorastos et al., 2019; Pervanidou et al., 2020).

Beyond the contributing effects of early life stress on child development and behavior, the stress system serves as a major adaptive physiological mechanism in living organisms. During the last decades, a plethora of studies have shown an altered activity (hyper- or hypo-activation) of the stress system, as evidenced by altered concentrations of stress-related biomarkers and brain changes in individuals with anxiety disorders, post-traumatic stress disorder (PTSD), and depression (Pervanidou, 2008). However, fewer data exist on the function of the stress system in children and adults with neurodevelopmental disorders [e.g., Autism, and Attention Deficit Hyperactivity Disorder (ADHD)] or traits related to altered neurodevelopment (e.g., deficits in attention or cognition). Individuals with Neurodevelopmental Disorders may exhibit non-typical function within the HPA axis and the LC-NE/ANS, both at the resting state and during the presence of social and/or other environmental stressors (Angeli et al., 2018; Anesiadou et al., 2021). In addition, they may appear with a greater percentage of mental (e.g., anxiety, low self-esteem) and physical co-morbidities (e.g., functional bowel symptoms) than the general population, which are relevant to altered stress mechanisms.

Prenatal stress as a contributing factor to neurodevelopmental impairment has been explored in three articles of this Research Topic. On the basis of a predisposing genetic background, environmental influences may affect brain development in its early stages, with a variety of potential effects. Potiris et al. have actually designed an animal model investigating the effects of prenatal food restriction on brain proteome in appropriately-grown and growth-restricted Wistar rats. This study, profiling 3,964 proteins, demonstrated that in both growth-restricted and non-growth-restricted neonates, a range of adaptive neurodevelopmental processes takes place, which may result in chronic stress, altered cellular morphology, and poor memory and learning outcomes. Furthermore, this study highlighted that it is prenatal food-restriction *per se* rather than birth weight that affects brain proteome, and this condition is potentially related to increased risk for impaired cognitive and developmental outcomes.

The study of Konstantakou et al. examined prospectively serum thyroid hormones and antibodies, measured at the 2nd and the 3rd trimester of gestation and at the first postpartum week, in association with measures of anxiety, depression and obsessive-compulsive disorder (OCD). The study demonstrated positive associations between low-normal thyroid function at the 2nd and 3rd trimesters of pregnancy and postpartum with anxiety, depression, and OCD scores. These findings are in close relation with the third article of this Research Topic, the mini-review examining the relations of prenatal maternal stress with thyroid function and neurodevelopment of the offspring, by Anifantaki et al. Indeed, both the HPA and the Hypothalamic-Pituitary-Thyroid (HPT) axes are involved in stress responses, whereas, their final effectors, the Glucocorticoids (GCs) and the Thyroid Hormones (THs), mediate several fundamental processes involved in neurodevelopment. It is well established that growth and neurodevelopment of the fetus depend on maternal hormones, especially during the first half of pregnancy. Altered concentrations of both GCs and THs can cause abnormalities in the neuronal and glial structures and functions, with subsequent effects on postnatal neurocognitive function. Experimental studies demonstrate that increased GC concentrations, related to maternal stress, may reduce maternal and, consequently, fetal circulating THs, either directly or through modifications in the expression of placental enzymes responsible for regulating fetal hormonal concentrations.

One opinion article of the Research Topic summarizes neuropharmacological aspects of stress. Konstandi et al. suggest that stress plays a central role in the regulation of drug bioavailability in the body, granted that pharmacotherapy may be a potential threat to homeostasis. Thus, stress may contribute to determining a drug's pharmacokinetic profile, as it regulates various enzymes that catalyze the metabolism of the majority of drugs.

Four articles of the Research Topic focus on peripheral neurobiological measures in children and adolescents. Makris et al. critically summarized the existing data on stress system alterations in children and adolescents with Autism Spectrum Disorder (ASD). This review focused on the variations of circadian rhythms of cortisol and alpha-amylase, as peripheral biomarkers of the HPA axis and LC-NE/ANS system, respectively, and on stress system responses to different stressors. This review article also included imaging and immunological findings that have been associated with stress system dysregulation in ASD youth. Finally, the article discussed the possible contribution of early life stress in ASD pathophysiology and the developmental trajectory of the stress system in ASD individuals. Similarly, Payen et al., in their systematic review, analyzed existing data on peripheral biomarkers of the gut and the heart in ADHD, the most prevalent NDD in childhood. The article provided a broader view of stress system components, including task-related heart rate reactivity (HRR) and gut microbiota data in children with and without ADHD. Lo Iacono et al., in their review, summarized evidence on the psychobiological effects of childhood sexual abuse, which has been considered a major traumatic experience in childhood. The authors provided data on specific alterations in the endocrine and immune systems, as well as data on epigenetic modifications related to child sexual abuse.

Finally, the research paper by Giannopoulos et al. investigated early life sensorimotor sex/age differences using Electroencephalographic (EEG) recordings to measure muscular and neural acoustic startle response (ASR) in a healthy young population. ASR is a cross-species indicator of sensorimotor and inhibitory mechanisms, showing distinct signature in cognitive aging, sex and psychopathological characterization. Neural ASR was assessed by two different analyses, Event-related Potentials (ERPs) and First-derivative Potentials (FDPs). In this study, the modulation of ASR by PPI and PPF was associated with biological sex and internal/external traits in childhood and adolescence, potentially useful to guide symptomatology and prevention of psychopathology.

The articles of this Research Topic highlighted the pivotal role of the stress system in neurodevelopment, either as a contributing environmental factor interacting with a genetic background, or as a major physiological mechanism of adaptation in individuals with neurodevelopmental disorders. Various neurobiological measures of stress have been analyzed and discussed by the authors, however, this is only a limited view of the role of the Stress System in the pathophysiology of NDDS. More data are needed to further elucidate and increase our knowledge on the longitudinal role of stress in brain development and in adaptation to everyday and novel/unexpected stressful stimuli.

## Author Contributions

PP wrote the initial draft. AA and GC critically reviewed the manuscript. All authors contributed to the article and approved the submitted version.

## Conflict of Interest

The authors declare that the research was conducted in the absence of any commercial or financial relationships that could be construed as a potential conflict of interest.

## Publisher's Note

All claims expressed in this article are solely those of the authors and do not necessarily represent those of their affiliated organizations, or those of the publisher, the editors and the reviewers. Any product that may be evaluated in this article, or claim that may be made by its manufacturer, is not guaranteed or endorsed by the publisher.
